# Lack of effect on in-hospital mortality of drugs used during COVID-19 pandemic: Findings of the retrospective multicenter COVOCA study

**DOI:** 10.1371/journal.pone.0256903

**Published:** 2021-09-14

**Authors:** Pia Clara Pafundi, Raffaele Galiero, Vittorio Simeon, Luca Rinaldi, Alessandro Perrella, Erica Vetrano, Alfredo Caturano, Maria Alfano, Domenico Beccia, Riccardo Nevola, Raffaele Marfella, Celestino Sardu, Carmine Coppola, Ferdinando Scarano, Paolo Maggi, Pellegrino De Lucia Sposito, Laura Vocciante, Carolina Rescigno, Costanza Sbreglia, Fiorentino Fraganza, Roberto Parrella, Annamaria Romano, Giosuele Calabria, Benedetto Polverino, Antonio Pagano, Carolina Bologna, Maria Amitrano, Vincenzo Esposito, Nicola Coppola, Nicola Maturo, Luigi Elio Adinolfi, Paolo Chiodini, Ferdinando Carlo Sasso, on behalf of COVOCA Study Group

**Affiliations:** 1 Department of Advanced Medical and Surgical Sciences, University of Campania “Luigi Vanvitelli”, Naples, Italy; 2 Medical Statistics Unit, Department of Physical and Mental Health and Preventive Medicine, University of Campania “Luigi Vanvitelli”, Naples, Italy; 3 Task Force Covid-19 Regione Campania, Italy; 4 Internal Medicine, Sant’Ottone Frangipane Hospital, ASL Avellino, Ariano Irpino (AV), Italy; 5 COVID Center "S. Anna e SS. Madonna della Neve" Hospital, Boscotrecase, Italy; 6 U.O.C. Infectious and Tropical diseases, S. Anna e S. Sebastiano Hospital, Caserta, Italy; 7 Covid Center - Maddaloni Hospital, Maddaloni, Italy; 8 General Medicine Unit, Loreto Mare Hospital, Naples, Italy; 9 U.O.C. Infectious Diseases and Neurology, Cotugno Hospital, Naples, Italy; 10 U.O.C. Infectious Diseases of the Elderly, Cotugno Hospital, Naples, Italy; 11 U.O.C. Anestesia and Intensive Care Unit, Cotugno Hospital, Naples, Italy; 12 U.O.C. Respiratory Infectious Diseases, Cotugno Hospital, Naples, Italy; 13 U.O.C. Pneumology, Moscati Hospital, Avellino, Italy; 14 IX^th^ Division of Infectious Diseases and Interventional Ultrasound, Cotugno Hospital, Naples, Italy; 15 "Giovanni da Procida" Hospital, Salerno, Italy; 16 Emergency and Acceptance Unit, "Santa Maria delle Grazie" Hospital, Pozzuoli, Italy; 17 Internal Medicine Unit, Ospedale Del Mare, Naples, Italy; 18 U.O.C. Internal Medicine - Moscati Hospital, Avellino, Italy; 19 IV^th^ Division of Immunodeficiency and Gender Infectious Diseases, Cotugno Hospital, Naples, Italy; 20 Department of Mental Health and Public Medicine, Centro COVID A.O.U. Vanvitelli, Naples, Italy; 21 U.O.S.D. Infectious Diseases Emergency and Acceptance, Cotugno Hospital, Naples, Italy; Ohio State University Wexner Medical Center Department of Surgery, UNITED STATES

## Abstract

**Introduction:**

During COVID-19 pandemic, the use of several drugs has represented the worldwide clinical practice. However, though the current increase of knowledge about the disease, there is still no effective treatment for the usage of drugs. Thus, we retrospectively assessed use and effects of therapeutic regimens in hospitalized patients on in-hospital mortality.

**Methods:**

COVOCA is a retrospective observational cohort study on 18 COVID centres throughout Campania Region Hospitals. We included adult patients with confirmed SARS-CoV-2 infection, discharged/dead between March/June 2020.

**Results:**

618 patients were included, with an overall in-hospital cumulative mortality incidence of 23.1%. Most prescribed early treatments were antivirals (72%), antibiotics (65%) and hydroxychloroquine/anticoagulants (≈50%). Tocilizumab, indeed, was largely prescribed late during hospitalization. Multivariable models, with a cut-off at day 2 for early COVID-19 therapy administration, did not disclose any significant association of a single drug administration on the clinical outcome.

**Discussion:**

COVOCA represents the first multicenter database in Campania region. None drug class used during the pandemic significantly modified the outcome, regardless of therapy beginning, both overall and net of those already in non-invasive ventilation (NIV)/ orotracheal intubation (OTI) at hospitalization. Our cumulative incidence of mortality seems lower than other described during the same period, particularly in Northern Italy.

## 1. Introduction

After the first outbreak of acute coronavirus-2 respiratory syndrome (SARS-CoV-2) reported in China in December of 2019 peak, named COVID-19 [1, 2], Italy was the first and most affected nation of the pandemic, officially declared by the WHO in March 2020 [3, 4]. Therefore, during the pandemic, Italian medical and political choices influenced other European nations and all over the world.

To date, no specific antiviral therapy has been identified yet. However, also in Italy the administration of monoclonal antibodies off-label has been recently approved, even though RCTs are few and still ongoing. The use of several drugs, usually in different associations, has represented the worldwide clinical practice and, more often, is still the first choice.

Antivirals (AVs), hydroxychloroquine (HyC), antibiotics (ATBs), Tocilizumab (mAbs), corticosteroids (CS) and low-molecular weight heparins (LMWH) have been the most frequently used drugs, usually accompanied by a supportive oxygen therapy.

During the course of pandemic, among all these drugs only corticosteroids, remdesivir and oxygen therapy seemed to determine a benefit in terms of both mortality and hospitalization rate reduction, though findings are still controversial [5–7]. At the beginning of the pandemic, indeed, due to the lack of evidence and guidelines, therapeutic regimens have been different among regions.

The most recent evidence has shown how Hydroxychloroquine, largely used during the first months of pandemic, actually is not effective against COVID-19, especially in the mild to moderate stages [8–10]. Similar findings were also reported in the case of a combined therapy with azithromycin [11, 12]. As well, Tocilizumab, largely used due to the initial encouraging outcomes after treatment, has not entirely demonstrated early results [0.83 hazard ratio for intubation or death as compared with the placebo group (95% confidence interval [CI], 0.38 to 1.81; P = 0.64), and 1.11 hazard ratio for disease worsening (95% CI, 0.59 to 2.10; P = 0.73)] [13, 14]. Controversial results have also been reported with corticosteroids (CS), even though, mostly during the early phase of inflammatory pulmonary damage, they have shown a good efficacy in the outcome’s improvement [15–17].

The improved knowledge about COVID-19 physiopathology, which have shown similarities with pulmonary edema, have stressed the importance of a supportive oxygen therapy, currently considered essential, mostly in mild to moderate disease stages.

Up to now, whether a therapeutic regimen is better than another has been poorly investigated.

However, though the current increase of knowledge about the disease, there is still no effective treatment and information about a proper timeline for the usage of drugs.

On these bases, we aimed to retrospectively assess the frequency of use of drugs, both as a single class and in association with each other, and the effects of therapeutic regimens started in hospitalized patients, classified according to WHO COVID-19 severity scale [18], on in-hospital mortality.

Originally, we also evaluated whether an early or delayed use of these drugs could determine different outcomes. Finally, we also verified the potential efficacy of different regimens of oxygen therapy in cases of respiratory distress.

## 2. Materials and methods

### 2.1 Study design and participants

COVOCA (observational study on the COVID-19 population hOspitalized in CAmpania Region) is a retrospective observational cohort study, which involved 18 COVID centres throughout Hospitals of Campania Region, Italy. This cohort of COVID-19 patients has already been presented and described in a previous paper [19].

Briefly, we included all adult patients (≥ 18 years) with laboratory confirmed SARS-CoV-2 infection, who completed their hospitalization (discharged or dead) in the period between March 13, 2020 and June 30, 2020, of whom clinical records were available. All data were fully anonymized by the participating centres before being accessed. The study was approved by the local Ethics Committee (University of Campania Luigi Vanvitelli) and is in accordance with 1976 Declaration of Helsinki and its later amendments.

### 2.2. Variables (outcome and exposure)

Microbiological diagnosis SARS-CoV-2 infection was defined by Real-Time Polymerase Chain Reaction of nasal-pharyngeal swab specimen. The outcome was in-hospital mortality, assessed either from data at discharge or death certificate.

Exposure variables were collected at hospital admission and have been extensively detailed in the first work regarding the cohort [19]. Overall, the following data were collected: demographic/anthropometric characteristics, anamnestic data, symptoms and previous comorbidities (smoking habit, diabetes, hypertension, chronic cardiac disease, chronic kidney disease (CKD), chronic liver disease (CLD), chronic respiratory disease, neurological disorders or malignancies). Furthermore, at physical examination, data on respiratory rates and Acute Respiratory Distress Syndrome (ARDS) Scale (defined as absent, mild, moderate and severe) were also collected. As for Glasgow Coma Score (GCS/15), this was categorized into: Mild impaired consciousness (GCS Score *>*13), Moderate/Severe impaired consciousness (GCS Score ≤13) and missing data. Also, were collected information on respiratory supports throughout the entire period of hospitalization (nasal cannula/Venturi Mask, non-invasive ventilation (NIV) and orotracheal intubation (OTI)) and classified in a Respiratory Severity Scale (RSS).

### 2.3. Drug therapy

Data on drug therapies, either ongoing or introduced during hospitalization, were widely collected.

Specifically, ongoing therapies such as Antihyperglycemics, Antihypertensives, Diuretics, Anticoagulants (Subcutaneous or Oral), Antiplatelets (Aspirin, Double Antiplatelet or Other), defined as baseline therapy reflect patients’ comorbid conditions. Instead, during hospitalization, specific therapies were introduced to counteract COVID pathology and specifically, in our cohort, they were as follows: Hydroxychloroquine, Anticoagulants, Antibiotics, Monoclonal Antibodies, Antivirals, Cortisone, Immunosuppressive, Antiplatelets, Paracetamol, NSAIDs, Plasma recovered, Immunoglobulins, Antiarrhythmics, Vasoactive, Inotropes, Crystalloids, Electrolytes, Albumin.

Particularly for most reported treatment and of interest in COVID-19 literature, in parallel to the description of whether they were used, a time-lag variable was created to identify patients undergoing either to an early or late treatment. This was done to avoid survivorship bias, considering that patients who live longer are more likely to receive a certain treatment/combination. Specifically, the time-lags variable (early/late) was categorized using a cut-off time (day-2). Day-2 represents treatments performed within the first two days since admission, considering day-0 as the time of hospitalization. Under this pattern, treatments were classified as: No treatment, early treatment (if occurred until day 2) and late treatment (from day 3 onwards). Moreover, a variable Days symptoms (difference between date of the beginning of symptoms and date of admission) related to lag variables for the use of each drug has been calculated.

### 2.4 Statistical analysis

Categorical data were expressed as number and percentages, whilst continuous variables as mean ± standard deviation (SD). The presence of missing data has been reported. Kendall’s τ_b_ coefficient was used to measure the ordinal association between specific COVID-19 treatments, as they were represented through an ordinal categorical variable: No Treatment, Early Treatment and Late Treatment. Kendall’s τ_b_ coefficient has been interpreted according to the following criteria: very weak if lower than ± 0.10, weak if from ± 0.10 to ± 0.19, moderate if from ± 0.20 to ± 0.29, strong if higher than ± 0.30. Multivariable logistic regression models were performed to evaluate association between in-hospital mortality and specific COVID-19 treatments. Drug treatments were evaluated in the model individually using the time-lag cut-off and adjusted according to previous findings [19]. Briefly, the selection had led to include as covariates age, sex, GCS/15 (mild/moderate/severe), Respiratory Severity Scale, Chronic Liver Disease, and Malignancies. Moreover, each model was fitted both on the whole population and on a subpopulation excluding patients without any type of oxygen therapy. Odds ratios and 95% confidence intervals (OR—95% CI) have been calculated for all models. A p-value *<*0.05 was considered as statistically significant. All analyses were performed using statistical software STATA v16 (StataCorp. 2019. College Station, TX: StataCorp LLC).

## 3. Results

### 3.1 Characteristics of the study population

662 patients positive at Sars-Cov-2 swab specimen, which required hospitalization, were considered eligible for the study and, of these, 44 were excluded due to incomplete clinical records. 618 patients were finally included in the study, mainly males (61.3%), with a mean age of 65 years (SD 15.2) and a median duration of hospitalization of 20 days [IQR 13–29 days]. The median time elapsed between onset of symptoms and hospitalization was of 4.5 days [IQR 2–7]. At the time of hospitalization, 63.6% of patients did not show any symptom of ARDS, while moderate and severe symptoms were observed, respectively, in about the 13.1% and 7.4%.

In addition, as for the RSS, 330 patients (53.4%) received respiratory support, at the time of hospitalization, either with Venturi mask or nasal cannula. Only the 13% needed either NIV or OTI. 46 patients (7.4%) showed moderate to severe impaired consciousness according to Glasgow Coma Scales (GCS/15).

On admission, almost half of the study population was under anti-hypertensive therapy (298; 48.2%) and the 19.6% took diuretics. Likewise, about the 20% underwent to anti-hyperglycemic therapy (119; 19.3%). As for antithrombotic therapy, indeed, the 16.3% already took anticoagulants, mostly subcutaneous (10%), whilst about was under antiplatelet therapy (19%), mostly aspirin (13.4%).

All clinical characteristics at admission are reported in Table 1.

**Table 1:** General characteristics of the study population (n = 618).

**Parameter**	
**Age,** mean (SD)	65 (15.2)
**Sex,** n (%)	
* M/F*	379 (61.3)/239 (38.7)
**GCS/15, n (%)**	
* Mild impaired consciousness*	448 (72.5)
* Moderate/Severe impaired consciousness*	46 (7.5)
* Missing*	124 (20.0)
**Respiratory Severity Scale,** n (%)	
* None*	211 (34.1)
* Mask/Glasses/Cannula*	330 (53.4)
* NIV*	48 (7.8)
* OTI*	29 (4.7)
**CLD,** n (%)	35 (5.7)
**Malignancies,** n (%)	53 (8.6)
**Therapy**	
**Antihyperglycemics**	
*Yes/No*	119 (19.3)/473 (76.5)
*Missing*	26 (4.2)
**Antihypertensives**	
*Yes/No*	298 (48.2)/292 (47.2)
*Missing*	28 (4.5)
**Diuretics**	
*Yes/No*	121 (19.6)/460 (74.4)
*Missing*	37 (6.0)
**Anticoagulants**	
*Subcutaneous*	62 (10.0)
*Oral*	39 (6.3)
*No*	455 (73.6)
*Missing*	62 (10.0)
**Antiplatelets**	
*Aspirin*	83 (13.4)
*Double Antiplatelet*	17 (2.8)
*Other*	17 (2.8)
*No*	501 (81.1)

**Abbreviations**: M: Male; F: Female; GCS: Glasgow Coma Score; RSS: Respiratory Severity Scale; NIV: Non-invasive ventilation; OTI: Orotracheal Intubation; CLD: Chronic Liver Disease (chronic hepatopathy from HCV and HBV, cirrhosis, non-alcoholic fatty liver disease (NAFLD)); SD: Standard Deviation; IQR: Interquartile Range

### 3.2 COVID-19 therapies

Given the focus on the therapeutic regimens specific for COVID-19 treatment, we first classified patients according to the administration of anyone among the following classes of drugs: corticosteroids, hydroxychloroquine, monoclonal antibodies (Tocilizumab), antibiotics, anticoagulants and antiviral (lopinavir/ritonavir). Patients were differentiated based on either an early (within the first two days since admission) and late administration (from day 3 in after).

**Fig 1:**
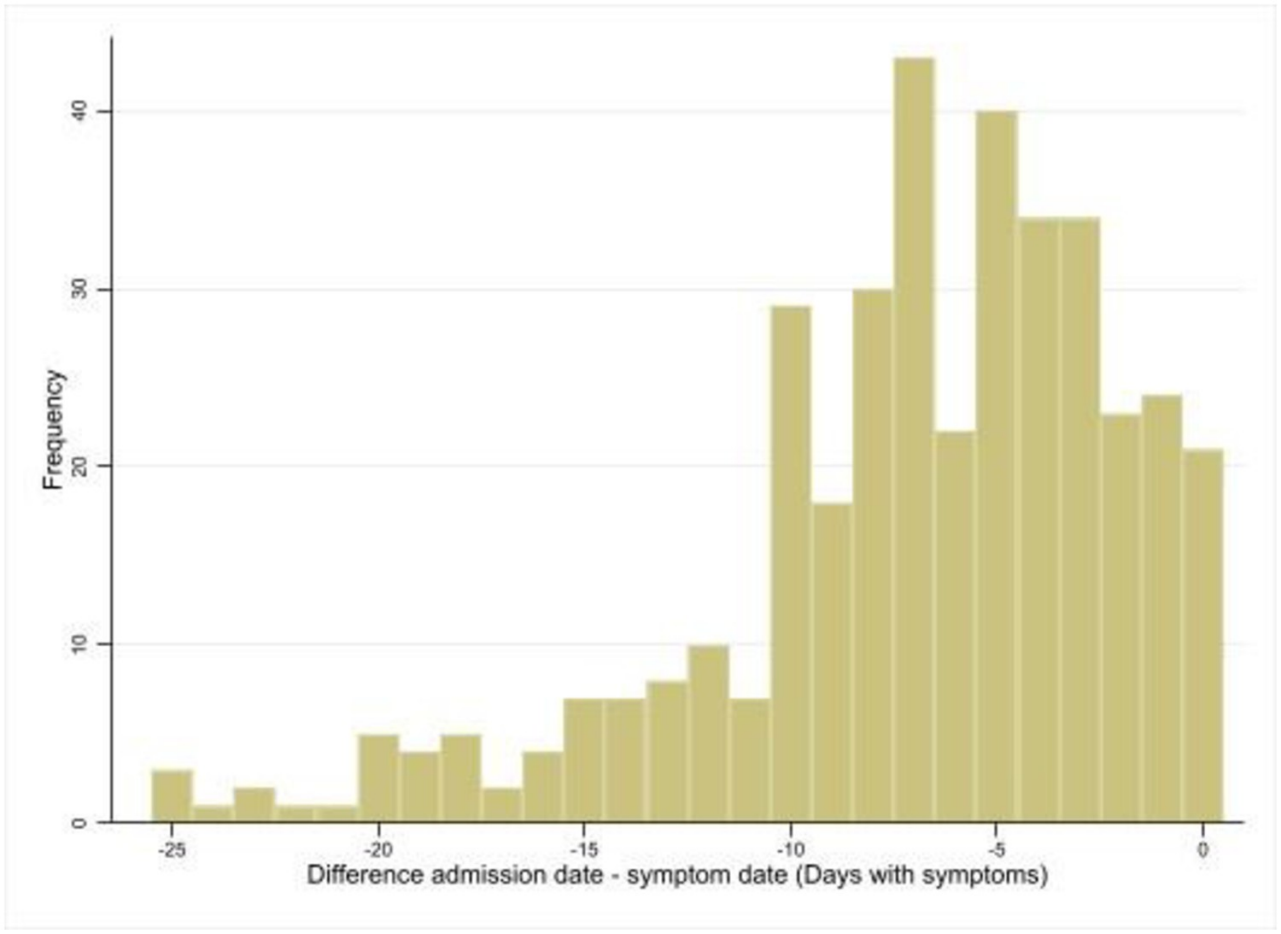
Different duration of illness prior to the admission (n = 385, 62.30% of COVOCA cohort).

**Fig 2:**
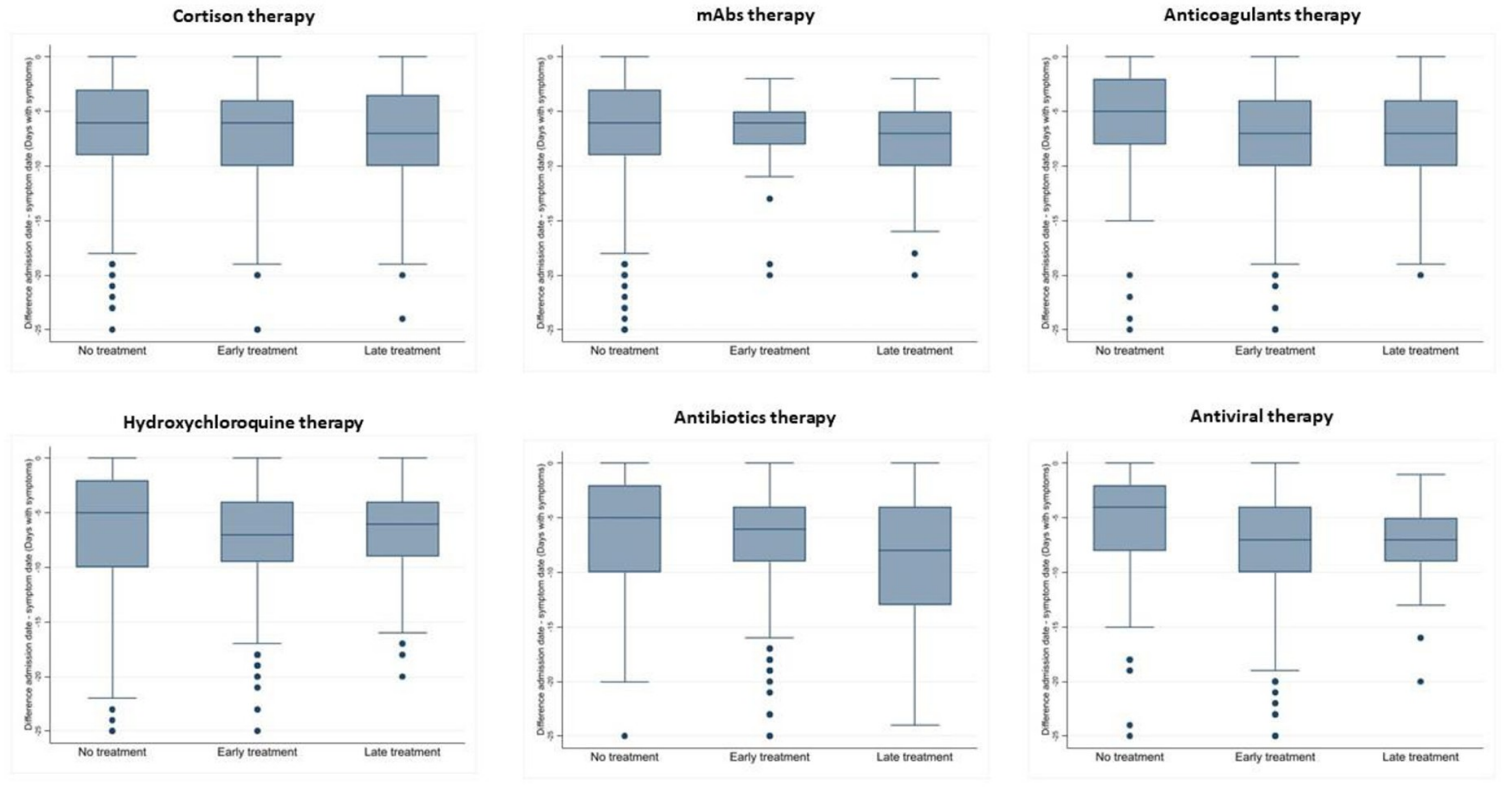
Variable days with symptoms (difference between date of admission and date of the beginning of symptoms) related to lag variables for the use of each drug. Comparison of days with symptoms related to lag variables for each treatment in analysis. Box and whisker plots were used in each panel. Box describes median and interquartile range; whiskers were represented using Tukey method and points describe outliers. The median and Interquartile Range [IQR] for each lag and treatment were reported. Kruskal-Wallis (KW) test, rank-based nonparametric test, was used to determine if there were statistically significant differences between lag variables group in each treatment. When the KW test was significant, post hoc analysis was performed using the Dunn’s (D) test with Bonferroni correction for multiple comparisons. P-value is reported. **Cortison therapy****:** No treatment -6 [-9, -3]; Early Treatment -6 [-10, -4]; Late Treatment -7 [-10, -3.5]; KW p = 0.47. **Hydroxychloroquine therapy****:** No treatment -5 [-10, -2]; Early Treatment -7 [-9.5, -4]; Late Treatment -6 [-9, -4]; KW p = 0.055. **mAbs therapy****:** No treatment -6 [-9, -3]; Early Treatment -6 [-8, -5]; Late Treatment -7 [-10, -5]; KW p = 0.24. **Antibiotics therapy****:** No treatment -5 [-10, -2]; Early Treatment -6 [-9, -4]; Late Treatment -8 [-13, -4]; KW p = 0.034; D—No Treat vs Early Treat p = 0.09, No Treat vs Late Treat p = 0.02, Early Treat vs Late Treat p = 0.21. **Anticoagulants therapy****:** No treatment -5 [-8, -2]; Early Treatment -7 [-10, -4]; Late Treatment -7 [-10, -4]; KW p = 0.011; D—No Treat vs Early Treat p = 0.02, No Treat vs Late Treat p = 0.01, Early Treat vs Late Treat p = 0.64. **Antiviral therapy****:** No treatment -4 [-8, -2]; Early Treatment -7 [-10, -4]; Late Treatment -7 [-9, -5]; KW p = 0.009; D—No Treat vs Early Treat p = 0.01, No Treat vs Late Treat p = 0.009, Early Treat vs Late Treat p = 0.44.

As reported in Table 2, the 72% of patients underwent to an early treatment with antivirals. Antibiotics were administered at beginning of hospitalization in the 65% of patients, whilst hydroxychloroquine and anticoagulants in almost half of the study cohort. Corticosteroid therapy administration was almost similar both early and late during hospitalization (20.9% and 15.8%, respectively), whilst monoclonal antibodies, especially Tocilizumab, were larger prescribed late during hospitalization. Different duration of illness prior to the admission has been calculated and reported in Fig 1, we recorded the longest duration with 25 days and a median of 6 days. Moreover, a median of duration of symptoms before the beginning of therapy has been calculated for each drug and reported in Fig 2. The statistical test (Kruskal-Wallis test) showed a difference between the time of administration (No, Early, Late) regarding days of symptoms in therapy with antibiotics (p = 0.034), anticoagulants (p = 0.011) and antivirals (p = 0.009). The test for multiple comparisons (Dunn’s test) shows that there is never a statistically significant difference in terms of lag time between Early and Late treatment while almost always the No treatment category are statistically significant compared to Early and Late with fewer days of symptoms before admission (Fig 2).

**Table 2:** COVID-19 therapies administered during hospitalization (n = 618).

**Parameter**	
**Hydroxychloroquine**, n (%)	
No	189 (30.6%)
early treatment	349 (56.5%)
late treatment	80 (12.9%)
**Anticoagulants**, n (%)	
No	202 (32.7%)
early treatment	325 (52.6%)
late treatment	91 (14.7%)
**Antibiotics,** n (%)	
No	153 (24.8%)
early treatment	402 (65.0%)
late treatment	63 (10.2%)
**Monoclonal Antibodies**, n (%)	
No	514 (83.2%)
early treatment	41 (6.6%)
late treatment	63 (10.2%)
**Antivirals**, n (%)	
No	107 (17.3%)
early treatment	445 (72.0%)
late treatment	66 (10.7%)
**Corticosteroids,** n (%)	
No	391 (63.3%)
early treatment	129 (20.9%)
late treatment	98 (15.8%)
**Immunosuppressive**, n (%)	5 (0.8%)
**Antiplatelets**, n (%)	
No	567 (91.7%)
Aspirin	38 (6.1%)
Double Antiplatelet	5 (0.8%)
Other	8 (1.3%)
**Paracetamol,** n (%)	176 (28.5%)
**NSAIDs,** n (%)	14 (2.3%)
**Plasma recovered,** n (%)	10 (1.6%)
**Immunoglobulins,** n (%)	9 (1.5%)
**Antiarrhythmics,** n (%)	13 (2.1%)
**Vasoactive,** n (%)	14 (2.3%)
**Inotropes,** n (%)	10 (1.6%)
**Crystalloids,** n (%)	99 (16.0%)
**Electrolytes,** n (%)	38 (6.1%)
**Albumin,** n (%)	33 (5.3%)
**Other drugs,** n (%)	72 (11.7%)

**Abbreviations**: NSAIDs: Nonsteroidal anti-inflammatory drugs.

**Other drugs**: chlorpheniramine, lenograstim, baricitinib, metoclopramide, everolimus, chloroquine, ruxolitinib, darbepoetin alfa, pentoxifylline, ambroxol, vilanterol.

** the time-lags variable (early/late) was categorized using a cut-off time (day-2). In depth, treatments were classified as: No treatment, early treatment (if occurred until day 2) and late treatment (from day 3 onwards).

We further assessed whether there was a drug-drug correlation related to the administration timeline by the Kendall’s Tau correlation coefficient for ordinal data. We observed only a few strong correlations (τ ≥ ±0.30). Particularly, the 48.2% (n = 298) were administered both antibiotics and hydroxychloroquine on the day of admission, with a τ = 0.43. Strong positive correlations, though slightly lower, were observed also for what concerned early administration of both anticoagulants/antivirals and hydroxychloroquine (τ = 0.38 and τ = 0.32, respectively), as well as for the early combination of antibiotics and anticoagulants (τ = 0.36). All data are described in S1 Table.

### 3.3 Impact of single drug therapy on in-hospital mortality

During the observation period, 143 in-hospital mortality events were recorded, with a cumulative incidence of 23.1%.

We fitted different multivariable models to test for the association between in-hospital mortality and each COVID-19 specific therapy, stratified according to the time of administration. The models were adjusted according to previous findings [19].

We fitted multivariable models, establishing a cut-off for the COVID-19 early therapy administration at day-2, and each model was fitted on the whole population as well as on a subpopulation obtained by excluding those who were without any respiratory support on admission.

All models did not disclose any significant association of a single drug administration on the clinical outcome of our COVID-19 study cohort, both overall and net of those without any respiratory support at hospitalization. Only a trend was observed as for early treatment administration of antivirals in this latter subpopulation (OR 2.07; 95%CI 0.94–4.58; p = 0.072). However, the analyses confirmed the previous significant associations between a poorer prognosis and male sex, chronic liver disease and malignancies. Likewise, NIV/OTI respiratory supports confirmed the independent association with a higher in-hospital mortality both overall and in the selected subpopulation. As well, moderate-to-severe impaired consciousness at GCS/15, revealed similar findings. All data are reported in Table 3 and in S2 Table. A specific model was fitted to evaluate the association between in-hospital mortality in patients under antiviral therapy with Ritonavir/Lopinavir, the most used antivirals drugs in our population. All data were reported in S3 Table.

**Table 3:** Association between in-hospital mortality and each COVID-19 specific therapy: Multivariate analysis* (n = 618).

	Whole sample (n = 618)			Excluding patients without any respiratory support (n = 407)		
	OR	95% CI	p	OR	95% CI	p
**Corticosteroids**						
No (ref.)						
early treatment	1.14	0.64–2.02	0.654	0.61	0.30–1.25	0.178
late treatment	1.38	0.76–2.51	0.291	0.92	0.46–1.85	0.822
**Hydroxychloroquine**						
No (ref.)						
early treatment	1.07	0.64–1.81	0.792	1.18	0.62–2.26	0.617
late treatment	1.19	0.58–2.46	0.633	1.19	0.49–2.85	0.700
**Anticoagulants**						
No (ref.)						
early treatment	1.44	0.83–2.48	0.190	1.20	0.63–2.29	0.584
late treatment	1.42	0.69–2.95	0.341	1.52	0.65–3.59	0.337
**Antibiotics**						
No (ref.)						
early treatment	1.08	0.63–1.83	0.784	0.87	0.47–1.61	0.651
late treatment	0.73	0.31–1.73	0.478	1.07	0.40–2.85	0.889
**Monoclonal Antibodies**						
No (ref.)						
early treatment	1.23	0.51–2.96	0.651	1.04	0.37–2.92	0.933
late treatment	1.51	0.74–3.06	0.254	1.24	0.56–2.77	0.599
**Antivirals**						
No (ref.)						
early treatment	1.36	0.73–2.54	0.331	2.07	0.94–4.58	0.072
late treatment	1.77	0.71–4.26	0.228	2.46	0.79–7.69	0.120

*adjusted by age, sex, GCS/15 (mild/moderate/severe), Respiratory Severity Scale, Chronic Liver Disease, Malignancies

** the time-lags variable (early/late) was categorized using a cut-off time (day-2). In depth, treatments were classified as: No treatment, early treatment (if occurred until day 2) and late treatment (from day 3 onwards).

## 4. Discussion

In our multicenter observational study, we evaluated the association between drug therapy and Covid-19 in-hospital mortality during the pandemic in the Campania region. Originally, we classified patients according to early therapy administration, within the first two days of hospitalization, and late therapy. We observed that none of the drug classes analyzed modified the outcome, both overall and net of those without any respiratory support at hospitalization. Moreover, as already reported, male gender, chronic liver disease, malignancies and NIV/OTI respiratory supports showed an independent association with a higher in-hospital mortality [19].

Notably, during the first peak, Lombardy was the most affected Italian region. Reports from the “Istituto Superiore della Sanità” (Italian National Health Institute) confirm Lombardy as the region with the highest mortality between March and May 2020 with 16,233 deaths (47.7% of all Italian victims of that period). Particularly, in-hospital mortality accounted for the 28.1% [20]. Similar findings were also observed in surrounding regions of Northern Italy. Campania, indeed, during the same period, experienced “only” 505 deaths from COVID-19 [21].

A nationwide retrospective Italian study on data retrieved from 160 hospitals charts between March and April 2020 showed, out of 1,397 COVID-19 patients hospitalized in Lombardy, a 35.5% in-hospital mortality incidence [22]. Conversely in the same period, a small cohort of 368 patients from different regions of southern Italy, reported cumulative incidence of mortality was 21.7% [22].

Currently, to the best of our knowledge, there is no registry on hospitalized COVID-19 patients in Campania, thus COVOCA represents the first multicenter database in this region. Intriguingly, our study shows a mortality cumulative incidence of 23%, lower than in most of Northern Italy.

Certainly, the largest spread of the virus in Northern Italy was of upmost stress for the Lombard Health System, which may have strongly affected COVID-19 in-hospital mortality. Especially during the first pandemic phase, in absence of clinical RCTs and scientific Societies recommendations, the therapeutic choices were center-dependent.

A study conducted at Luigi Sacco Hospital, Milan, between February 21 and April, 30 2020, reported a very high risk of potential adverse events due to drug-drug interactions (DDIs) in COVID-19 patients. This result was consistent with INTERcheck^®^, a Computerized Prescription Support System (CPSS) developed by the Pharmacological Research Institute Mario Negri (IRCCS) of Milan (Italy) to improve the appropriateness of prescriptions. Currently, 88% of hospitalized Covid-19 patients had an increased risk of cardiotoxicity (QT prolongation, torsade de Pointes or life-threatening arrhythmias) due to in hospital treatment [23]. In particular, a dramatic increase in the number of potentially severe DDIs was mostly observe with the concomitant treatment with hydroxychloroquine and lopinavir/ritonavir.

A retrospective study performed between March, 13 to April, 3 2020 at Niguarda Hospital, Milan has shown, according to the regional recommendations, a use of hydroxychloroquine, lopinavir/ritonavir and remdesivir as standard of care in 89.9%, 85.1% and 8.1% of COVID-19 patients, respectively [24]. These findings confirm the large use, during the pandemic of COVID-19 pandemic, of the association of drugs potentially triggering severe DDIs.

In the COVOCA study, hydroxychloroquine and any antiviral drugs were used in 69.4% and 82.7% of patients, respectively. Moreover, 17.3% of COVID-19 patients did not receive any association of hydroxychloroquine with an antiviral drug, neither early nor late during hospitalization.

Therefore, our study cohort seems at lower risk of DDIs than the aforementioned populations from Northern Italy studies. The most brutal impact of the first pandemic peak in these regions has more likely led doctors to a more aggressive pharmacological attitude, with a consequent increase in serious adverse events due to dangerous drug associations.

Interestingly, most recent evidence has reported that in an early phase of disease, characterized by the beginning of the cytokines’ cascade, antimalarial drugs could be effective in its prevention, whilst monoclonal antibodies could better act in a more delayed phase, when the cascade is active [25].

Due to this evidence, we considered two in-hospital subpopulations, one for an early administration of drugs and a second with a late treatment. However, no significant difference emerged between the two subgroups. We could assume that lack of significance in the case of hydroxychloroquine is ascribable to a use in a more advanced phase of disease, likely due to the time between beginning of symptoms and hospitalization.

Likewise, corticosteroids, particularly dexamethasone, are recommended, especially if used after the first week of disease, when the inflammatory phase reaches the peak, and there is a need to initiate respiratory assistance [26]. However, data on their efficacy are controversial. The RECOVERY study, for example, although demonstrating the efficacy of dexamethasone in terms of 28-days mortality reduction, shows no efficacy in patients not under any respiratory support. Actually, in our paper we observed no efficacy of corticosteroids therapy on mortality, both in patients under mechanical ventilation and not [17].

The test for multiple comparisons showed that for all treatments the lag time is not significantly different between the Early and Late treatment groups. Intriguingly, for some drugs the lag time is significantly shorter in the no treatment group compared to both the Late and Early treatment groups. It can be hypothesized that patients in the no treatment group were hospitalized early and thus they did not require treatment, or all available drugs.

This study has several limitations. First, the observational design precludes the study from defining a certain cause-effect relationship. On the other hand, numerous RCTs over the last year have ruled out a protective role against COVID-19 mortality for different drug classes, thus confirming our real-life data. Second, due to the observational nature of the study, we cannot exclude the presence of confounding by indication, very common in this type of study. This bias could occur in relation to both beneficial and harmful outcomes and may lead to either an increase or decrease in the apparent risk of the outcome [27]. The lack of a survival bias effect in the late treated patients supports the presence of this indication bias. Third, inpatient therapy was analysed for drug classes and not for single molecules. However, our results are not with certainty attributable to all drugs in each class. Moreover, most of centres were unable to provide BMI data due to patients’ clinical conditions.

## 5. Conclusions

This retrospective observational study conducted on 18 COVID Centers is the first to report both management and in-hospital mortality in Campania region. According to our findings, no drug class used during the pandemic, significantly changed mortality risk, regardless of therapy beginning. The cumulative incidence of mortality in this setting seems lower than that described during the same period in other series, particularly in northern Italy. This difference could be due to the different association of drugs at a higher risk for DDIs. Further pharmacovigilance studies are needed to clarify this hypothesis.

## Supporting information

S1 Table Drug-drug correlation related to the administration timeline by the Kendall’s Tau correlation coefficient. (DOCX)

S2 Table Association between in-hospital mortality and each COVID-19 specific therapy: Multivariable analysis. (DOCX)

S3 Table Association between in-hospital mortality in patients under antiviral therapy with Ritonavir/Lopinavir. (DOCX)

## References

[1] WHO. “Clinical management of COVID-19 disease”. REFERENCE NUMBER: WHO/2019-nCoV/clinical/2020.5. https://apps.who.int/iris/bitstream/handle/10665/332196/WHO-2019-nCoV-clinical-2020.5-eng.pdf?sequence=1&isAllowed=y (last accessed on March 13, 2021)

[2] Valdenassi L, Franzini M, Ricevuti G, Rinaldi L, Galoforo AC, Tirelli U. Potential mechanisms by which the oxygen-ozone (O2-O3) therapy could contribute to the treatment against the coronavirus COVID-19. Eur Rev Med Pharmacol Sci 2020;24:4059–61 doi: 10.26355/eurrev_202004_20976 32374009

[3] Carta MG, Scano A, Lindert J, Bonanno S, Rinaldi L, Fais S, et al. Association between the spread of COVID-19 and weather-climatic parameters. Eur Rev Med Pharmacol Sci. 2020;24:8226–8231 doi: 10.26355/eurrev_202008_22512 32767354

[4] Iavarone M, D’Ambrosio R, Soria A, Triolo M, Pugliese N, Del Poggio P, et al. High rates of 30-day mortality in patients with cirrhosis and COVID-19. J Hepatol.;73(5):1063–1071. 10.1016/j.jhep.2020.06.001 32526252 PMC7280108

[5] Jiang B, Wei H. Oxygen therapy strategies and techniques to treat hypoxia in COVID-19 patients. Eur Rev Med Pharmacol Sci.;24(19):10239–10246. 10.26355/eurrev_202010_23248 33090435 PMC9377789

[6] Wang Y, Zhang D, Du G, Du R, Zhao J, Jin Y, et al. Remdesivir in adults with severe COVID-19: a randomised, double-blind, placebo-controlled, multicentre trial. Lancet.;395(10236):1569–1578. doi: 10.1016/S0140-6736(20)31022-9 32423584 PMC7190303

[7] Ramiro S, Mostard RLM, Magro-Checa C, van Dongen CMP, Dormans T, Buijs J, et al. Historically controlled comparison of glucocorticoids with or without tocilizumab versus supportive care only in patients with COVID-19-associated cytokine storm syndrome: results of the CHIC study. Ann Rheum Dis. Sep;79(9):1143–1151. 10.1136/annrheumdis-2020-218479 32719045 PMC7456552

[8] Tang W, Cao Z, Han M, Wang Z, Chen J, Sun W et al. Hydroxychloroquine in patients with mainly mild to moderate coronavirus disease 2019: open label, randomised controlled trial. BMJ.;369:m1849. 10.1136/bmj.m1849 32409561 PMC7221473

[9] Borba MGS, Val FFA, Sampaio VS, Alexandre MAA, Melo GC, Brito M, et al. Effect of High vs Low Doses of Chloroquine Diphosphate as Adjunctive Therapy for Patients Hospitalized With Severe Acute Respiratory Syndrome Coronavirus 2 (SARS-CoV-2) Infection: A Randomized Clinical Trial. JAMA Netw Open;3(4):e208857. 10.1001/jamanetworkopen.2020.8857 32330277 PMC12124691

[10] Patel TK, Barvaliya M, Kevadiya BD, Patel PB, Bhalla HL. Does Adding of Hydroxychloroquine to the Standard Care Provide any Benefit in Reducing the Mortality among COVID-19 Patients?: a Systematic Review. J Neuroimmune Pharmacol.;15(3):350–358. 10.1007/s11481-020-09930-x 32519281 PMC7280684

[11] Omrani AS, Pathan SA, Thomas SA, Harris TRE, Coyle PV, Thomas CE, et al. Randomized double-blinded placebo-controlled trial of hydroxychloroquine with or without azithromycin for virologic cure of non-severe Covid-19. EClinicalMedicine.;29:100645. 10.1016/j.eclinm.2020.100645 33251500 PMC7678437

[12] Cavalcanti AB, Zampieri FG, Rosa RG, Azevedo LCP, Veiga VC, Avezum A, et al. Hydroxychloroquine with or without Azithromycin in Mild-to-Moderate Covid-19. N Engl J Med.;383(21):2041–2052. 10.1056/NEJMoa2019014 32706953 PMC7397242

[13] Keske Ş, Tekin S, Sait B, İrkören P, Kapmaz M, Çimen C, et al. Appropriate use of tocilizumab in COVID-19 infection. Int J Infect Dis.;99:338–343. 10.1016/j.ijid.2020.07.036 32726724 PMC7382959

[14] Stone JH, Frigault MJ, Serling-Boyd NJ, Fernandes AD, Harvey L, Foulkes AS, et al. Efficacy of Tocilizumab in Patients Hospitalized with Covid-19. N Engl J Med.;383(24):2333–2344. 10.1056/NEJMoa2028836 33085857 PMC7646626

[15] Ye Z, Wang Y, Colunga-Lozano LE, Prasad M, Tangamornsuksan W, Rochwerg B, et al. Efficacy and safety of corticosteroids in COVID-19 based on evidence for COVID-19, other coronavirus infections, influenza, community-acquired pneumonia and acute respiratory distress syndrome: a systematic review and meta-analysis. CMAJ.;192(27):E756–E767. 10.1503/cmaj.200645 32409522 PMC7828900

[16] Zha L, Li S, Pan L, Tefsen B, Li Y, French N, et al. Corticosteroid treatment of patients with coronavirus disease 2019 (COVID-19). Med J Aust.;212(9):416–420. 10.5694/mja2.50577 32266987 PMC7262211

[17] Horby P, Lim WS, Emberson JR, Mafham M, Bell JL, Linsell L, et al. Dexamethasone in Hospitalized Patients with Covid-19. N Engl J Med.;384(8):693–704. 10.1056/NEJMoa2021436 32678530 PMC7383595

[18] Guan WJ, Liang WH, Zhao Y, Liang HR, Chen ZS, Li YM, et al. Comorbidity and its impact on 1590 patients with COVID-19 in China: a nationwide analysis. Eur Respir J.;55(5):2000547. 10.1183/13993003.00547-2020 32217650 PMC7098485

[19] Galiero R, Pafundi PC, Simeon V, Rinaldi L, Perrella A, Vetrano E, et al. Impact of chronic liver disease upon admission on COVID-19 in-hospital mortality: Findings from COVOCA study. PLoS One.;15(12):e0243700. 10.1371/journal.pone.0243700 33301529 PMC7728173

[20] Ferroni E, Giorgi Rossi P, Spila Alegiani S, Trifirò G, Pitter G, Leoni O, et al. Survival of Hospitalized COVID-19 Patients in Northern Italy: A Population-Based Cohort Study by the ITA-COVID-19 Network. Clin Epidemiol.;12:1337–1346. 10.2147/CLEP.S271763 33335428 PMC7737545

[21] Impatto dell’epidemia COVID-19 sulla mortalità totale della popolazione residente anno 2020, 5 marzo 2021, Istituto Superiore della Sanità –ISTAT. https://www.istat.it/it/files//2021/03/Report_ISS_Istat_2020_5_marzo.pdf?fbclid=IwAR0HjS9VEgZFCBrLKEPNAkvvZVIFvvBUr2Lon2-TJu8qYfg6ScVqgPmt_6A (last accessed March 13, 2021)

[22] De Rosa S, Spaccarotella C, Basso C, Calabrò MP, Curcio A, Filardi PP, et al. Reduction of hospitalizations for myocardial infarction in Italy in the COVID-19 era. Eur Heart J.;41(22):2083–2088. 10.1093/eurheartj/ehaa409 32412631 PMC7239145

[23] Cattaneo D, Pasina L, Maggioni AP, Giacomelli A, Oreni L, Covizzi A, et al. Drug-Drug Interactions and Prescription Appropriateness in Patients with COVID-19: A Retrospective Analysis from a Reference Hospital in Northern Italy. Drugs Aging.;37(12):925–933. 10.1007/s40266-020-00812-8 33150470 PMC7641655

[24] Rossotti R, Travi G, Ughi N, Corradin M, Baiguera C, Fumagalli R, et al. Safety and efficacy of anti-il6-receptor tocilizumab use in severe and critical patients affected by coronavirus disease 2019: A comparative analysis. J Infect.;81(4):e11–e17. 10.1016/j.jinf.2020.07.008 32652164 PMC7345400

[25] Valentini M, Zmerly H. Antirheumatic drugs for COVID-19 treatment based on the phases of the disease: Current concept. J Popul Ther Clin Pharmacol.;27(S Pt 1):e14–e25. 10.15586/jptcp.v27iSP1.689 32650355

[26] Tortajada C, Colomer E, Andreu-Ballester JC, Esparcia A, Oltra C, Flores J. (2021) Corticosteroids for COVID-19 patients requiring oxygen support? Yes, but not for everyone: Effect of corticosteroids on mortality and intensive care unit admission in patients with COVID-19 according to patients’ oxygen requirements. J Med Virol.;93(3):1817–1823. 10.1002/jmv.26635 33107607

[27] Catalogue of bias collaboration, Aronson JK, Bankhead C, Mahtani KR, Nunan D. Confounding by indication. In Catalogue Of Biases. 2018. https://catalogofbias.org/biases/confounding-by-indication (last accessed on March 13, 2021)

