# Supplementary material for: Lack of effect on in-hospital mortality of drugs used during COVID-19 pandemic: Findings of the retrospective multicenter COVOCA study
Source: PLoS One. 2021 Sep 14;16(9):e0256903. doi: 10.1371/journal.pone.0256903 (PMC8439483; doi:10.1371/journal.pone.0256903)
Supplement: S1 Table — (DOCX) [file pone.0256903.s001.docx]

| **S1 Table.** Drug-drug correlation related to the administration timeline by the Kendall’s Tau correlation coefficient. | | | | | | | | | | | | | | | | | | | |
| --- | --- | --- | --- | --- | --- | --- | --- | --- | --- | --- | --- | --- | --- | --- | --- | --- | --- | --- | --- |
|  |  | **Corticosteroids** | | | **Hydroxychloroquine** | | | **mAbs** | | | **Antibiotics** | | | **Anticoagulants** | | | **Antivirals** | | |
|  |  | **No** | **Early** | **Late** | **No** | **Early** | **Late** | **No** | **Early** | **Late** | **No** | **Early** | **Late** | **No** | **Early** | **Late** | **No** | **Early** | **Late** |
| **Corticosteroids** | **N** |  | | | 151 | 195 | 45 | 345 | 15 | 31 | 126 | 225 | 40 | 162 | 180 | 49 | 61 | 291 | 39 |
|  | **E** |  |  |  | 23 | 97 | 9 | 93 | 18 | 18 | 16 | 110 | 3 | 20 | 100 | 9 | 27 | 96 | 6 |
|  | **L** |  |  |  | 15 | 57 | 26 | 76 | 8 | 14 | 11 | 67 | 20 | 20 | 45 | 33 | 19 | 58 | 21 |
| **Hydroxychloroquine** | **N** | 0.21 | | |  | | | 173 | 7 | 9 | 111 | 59 | 19 | 117 | 62 | 10 | 49 | 133 | 7 |
|  | **E** |  |  |  |  |  |  | 274 | 32 | 43 | 35 | 298 | 16 | 61 | 246 | 42 | 51 | 287 | 11 |
|  | **L** |  |  |  |  |  |  | 67 | 2 | 11 | 7 | 45 | 28 | 24 | 17 | 39 | 7 | 25 | 48 |
| **mAbs** | **N** | 0.15 | | | 0.11 | | |  | | | 142 | 317 | 55 | 182 | 259 | 73 | 100 | 362 | 52 |
|  | **E** |  |  |  |  |  |  |  |  |  | 6 | 34 | 1 | 6 | 29 | 6 | 2 | 37 | 2 |
|  | **L** |  |  |  |  |  |  |  |  |  | 5 | 51 | 7 | 14 | 37 | 12 | 5 | 46 | 12 |
| **Antibiotics** | **N** | 0.18 | | | 0.43 | | | 0.10 | | |  | | | 101 | 44 | 8 | 30 | 116 | 7 |
|  | **E** |  |  |  |  |  |  |  |  |  |  |  |  | 81 | 268 | 53 | 67 | 295 | 40 |
|  | **L** |  |  |  |  |  |  |  |  |  |  |  |  | 20 | 13 | 30 | 10 | 34 | 19 |
| **Anticoagulants** | **N** | 0.22 | | | 0.38 | | | 0.11 | | | 0.36 | | |  | | | 39 | 137 | 26 |
|  | **E** |  |  |  |  |  |  |  |  |  |  |  |  |  |  |  | 58 | 255 | 12 |
|  | **L** |  |  |  |  |  |  |  |  |  |  |  |  |  |  |  | 10 | 53 | 28 |
| **Antivirals** | **N** | 0.00 | | | 0.32 | | | 0.11 | | | 0.12 | | | 0.09 | | |  | | |
|  | **E** |  |  |  |  |  |  |  |  |  |  |  |  |  |  |  |  |  |  |
|  | **L** |  |  |  |  |  |  |  |  |  |  |  |  |  |  |  |  |  |  |
