# Supplementary material for: Lack of effect on in-hospital mortality of drugs used during COVID-19 pandemic: Findings of the retrospective multicenter COVOCA study
Source: PLoS One. 2021 Sep 14;16(9):e0256903. doi: 10.1371/journal.pone.0256903 (PMC8439483; doi:10.1371/journal.pone.0256903)
Supplement: S2 Table — (DOCX) [file pone.0256903.s002.docx]

**S2 Table.** Association between in-hospital mortality and each COVID-19 specific therapy: multivariable analysis.

| Whole sample |  | **Corticosteroids** | **Hydroxychloroquine** | **Anticoagulants** | **Antibiotics** | **Monoclonal Antibodies** | **Antivirals** |
| --- | --- | --- | --- | --- | --- | --- | --- |
|  | **Age** | 1.05 (1.03,1.06) | 1.05 (1.03,1.06) | 1.04 (1.03,1.06) | 1.05 (1.03,1.06) | 1.05 (1.03,1.07) | 1.05 (1.03,1.07) |
|  | **Sex** |  |  |  |  |  |  |
|  | *F (ref.)* | 1 | 1 | 1 | 1 | 1 | 1 |
|  | *M* | 1.78 (1.09,2.91) | 1.78 (1.09,2.92) | 1.75 (1.07,2.87) | 1.77 (1.08,2.90) | 1.7 (1.03,2.79) | 1.76 (1.08,2.88) |
|  | **GCS/15** |  |  |  |  |  |  |
|  | *Mild impaired consciousness (ref.)* | 1 | 1 | 1 | 1 | 1 | 1 |
|  | *Moderate/Severe impaired consciousness* | 7.53 (3.56,15.94) | 7.58 (3.61,15.93) | 7.78 (3.69,16.39) | 7.52 (3.58,15.79) | 7.43 (3.53,15.63) | 7.49 (3.56,15.76) |
|  | *Missing* | 1.96 (1.12,3.42) | 1.94 (1.11,3.40) | 2.03 (1.16,3.54) | 1.99 (1.14,3.47) | 1.93 (1.11,3.33) | 1.83 (1.05,3.18) |
|  | **Respiratory Severity Scale** |  |  |  |  |  |  |
|  | *None (ref.)* | 1 | 1 | 1 | 1 | 1 | 1 |
|  | *Mask/Glasses/Cannula* | 1.23 (0.72,2.12) | 1.26 (0.74,2.16) | 1.29 (0.75,2.22) | 1.27 (0.74,2.17) | 1.23 (0.72,2.11) | 1.26 (0.74,2.17) |
|  | *NIV* | 5.51 (2.48,12.22) | 5.64 (2.55,12.47) | 5.73 (2.60,12.63) | 5.84 (2.64,12.90) | 5.35 (2.40,11.93) | 5.5 (2.48,12.18) |
|  | *OTI* | 6.82 (2.03,22.67) | 7.19 (2.15,22.79) | 6.07 (1.69,20.05) | 7.46 (2.28,23.30) | 6.79 (2.01,21.64) | 8.44 (2.60,26.23) |
|  | **CLD** | 5.69 (2.53,12.77) | 5.64 (2.52,12.64) | 5.41 (2.42,12.12) | 5.82 (2.60,13.01) | 5.76 (2.55,12.99) | 5.51 (2.45,12.38) |
|  | **Malignancies** | 2.37 (1.19,4.74) | 2.45 (1.23,4.90) | 2.34 (1.17,4.66) | 2.53 (1.27,5.07) | 2.43 (1.22,4.86) | 2.47 (1.24,4.94) |
| Excluding patients without any respiratory support | **Age** | 1.05 (1.02,1.07) | 1.05 (1.02,1.07) | 1.05 (1.02,1.07) | 1.04 (1.02,1.07) | 1.05 (1.02,1.07) | 1.05 (1.03,1.07) |
|  | **Sex** |  |  |  |  |  |  |
|  | *F (ref.)* | 1 | 1 | 1 | 1 | 1 | 1 |
|  | *M* | 1.83 (1.00,3.35) | 1.87 (1.02,3.43) | 1.81 (0.99,3.31) | 1.84 (1.01,3.36) | 1.81 (0.99,3.33) | 1.81 (0.99,3.32) |
|  | **GCS/15** |  |  |  |  |  |  |
|  | *Mild impaired consciousness (ref.)* | 1 | 1 | 1 | 1 | 1 | 1 |
|  | *Moderate/Severe impaired consciousness* | 10.6 (4.22,26.57) | 9.59 (3.90,23.54) | 9.92 (4.03,24.45) | 9.64 (3.91,23.75) | 9.47 (3.85,23.29) | 9.61 (3.89,23.74) |
|  | *Missing* | 2.53 (1.28,4.98) | 2.86 (1.43,5.74) | 2.77 (1.41,5.45) | 2.66 (1.35,5.23) | 2.72 (1.39,5.32) | 2.42 (1.22,4.78) |
|  | **Respiratory Severity Scale** |  |  |  |  |  |  |
|  | *Mask/Glasses/Cannula (ref)* | 1 | 1 | 1 | 1 | 1 | 1 |
|  | *NIV* | 5.02 (2.39,10.55) | 4.94 (2.35,10.40) | 4.97 (2.36,10.47) | 4.98 (2.37,10.47) | 4.94 (2.33,10.44) | 4.77 (2.26,10.05) |
|  | *OTI* | 10.61 (4.27,26.41) | 10.22 (4.11,25.44) | 10.2 (4.03,25.84) | 10.57 (4.27,26.12) | 10.52 (4.25,26.04) | 13.07 (5.06,33.77) |
|  | **CLD** | 11.8 (4.22,32.98) | 11.22 (4.02,31.33) | 10.84 (3.89,30.18) | 11.57 (4.14,32.32) | 11.57 (4.13,32.39) | 11.01 (3.93,30.84) |
|  | **Malignancies** | 2.44 (1.03,5.77) | 2.54 (1.06,6.06) | 2.47 (1.04,5.87) | 2.44 (1.03,5.80) | 2.48 (1.04,5.89) | 2.66 (1.10,6.40) |
| Data are reported as OR (95%CI). The full model, excluding information on treatment, is presented. Each column describes a single multivariable model for specific treatment. Estimates are reported also for subpopulation in study. Abbreviations: M: Male; F: Female; GCS: Glasgow Coma Score; RSS: Respiratory Severity Scale; CLD: Chronic Liver Disease; NIV: Non-invasive ventilation; IOT: Orotracheal Intubation; OR: Odds Ratio; CI: Confidence Interval. | | | | | | | |
