# Supplementary material for: Lack of effect on in-hospital mortality of drugs used during COVID-19 pandemic: Findings of the retrospective multicenter COVOCA study
Source: PLoS One. 2021 Sep 14;16(9):e0256903. doi: 10.1371/journal.pone.0256903 (PMC8439483; doi:10.1371/journal.pone.0256903)
Supplement: S3 Table — (DOCX) [file pone.0256903.s003.docx]

**S3 Table.** Association between in-hospital mortality in patients under Antiviral therapy with Ritonavir/Lopinavir.

| **Subgroup only with patients under Ritonavir/Lopinavir therapy (n=372)** | | | |
| --- | --- | --- | --- |
|  | **OR** | **95% CI** | **P** |
| **Antiviral logistic** |  |  |  |
| *Early treatment (ref.)* | 1 | - | - |
| *Late treatment* | 1.45 | 0.60-3.47 | 0.40 |

| **Subgroup with patients under Ritonavir/Lopinavir therapy and patients without any antiviral therapy (n=479)** | | | |
| --- | --- | --- | --- |
|  | **OR** | **95% CI** | **P** |
| **Antiviral logistic**  *No treatment*  *Early treatment*  *Late treatment* | 1  1.09  1.57 | -  0.57-2.05  0.58-4.25 | -  0.79  0.37 |
| *adjusted by age, sex, GCS/15 (mild/moderate/severe), Respiratory Severity Scale, Chronic Liver Disease, Malignancies  ** the time-lags variable (early/late) was categorized using a cut-off time (day-2). | | | |
